# Platelets from early-stage Alzheimer patients show enhanced amyloid binding, an elevated open canalicular system and sex-specific differences in their activation profile

**DOI:** 10.3389/fneur.2026.1759268

**Published:** 2026-03-19

**Authors:** Lili Donner, Julia Christl, Milenko Kujovic, Tillmann Supprian, Margitta Elvers

**Affiliations:** 1 Clinic of Vascular and Endovascular Surgery, Medical Faculty and University Hospital Duesseldorf, Heinrich-Heine-University, Düsseldorf, Germany; 2 Department of Psychiatry, Medical Faculty, LVR-Clinic Düsseldorf, Heinrich-Heine University, Düsseldorf, Germany

**Keywords:** Alzheimer's disease, open canalicular system, platelet activation, platelets, ß-amyloid

## Abstract

**Introduction:**

Alzheimer's disease (AD) is associated with neurodegeneration and dementia. Key clinical hallmarksinclude the deposition of amyloid-ß (Aβ) into senile plaques in the brain parenchyma and in cerebral vessels known as cerebral amyloid angiopathy (CAA). Currently, anti-Aß antibodies are emerging as possible therapy for AD. Several biomarkers, such as Aß and tau-protein have gained diagnostic relevance for early AD; however, their assessment requires cerebrospinal fluid. Therefore, blood-based biomarkers for AD screening are urgently needed.

**Methods:**

Patients diagnosed with early AD were analyzed for extracellular Aß binding to platelets, platelet morphology and platelet activation, and were compared with age-matched controls.

**Results:**

Platelet number and size were unaltered between groups. However, platelets isolated from AD patients exhibited increased surface APP/Aβ immunoreactivity compared with age-matched controls. Transmission electron microscopy revealed altered platelet morphology in AD patients, including changes in the number of dense granules and an increased area of the open canalicular system (OCS). While only minor differences in platelet activation were detected between patients and controls, a significant reduction in integrin αIIbβ3 (fibrinogen receptor) activation was observed in platelets from female compared to male AD patients, as determined by flow cytometry.

**Conclusion:**

The results presented here emphasize the importance of understanding whether platelets contribute to AD pathology in a sex-specific manner. Furthermore, platelet parameters may serve as promising biomarker for early AD prognosis, as platelets are easily accessible via blood sampling. These parameters may include sex-specific platelet activation profiles, the ability of platelets to bind APP/Aß at their surface, and OCS dimensions assessed by electron microscopy.

## 1 Introduction

Alzheimer's disease (AD) is a neurodegenerative disorder characterized by a progressive cognitive decline (1). Patients with AD exhibit a distinct neuropathological profile, including extracellular deposition of amyloid-ß (Aß) in senile plaques and the formation of intracellular neurofibrillary tangles composed of hyperphosphorylated tau protein (2). In addition, approximately 80% of AD patients develop cerebral amyloid angiopathy (CAA) which includes the accumulation and aggregation of Aß in cerebral vessels (3, 4). Beyond Aß and tau pathology, a cascade of pathophysiological events, including neuroinflammation and blood-brain-barrier dysfunction (4), contributes to neuronal loss and cognitive decline in AD.

Currently, around 57 million people worldwide suffer from dementia (5). However, with increasing age of the global population and the emergence of potentially effective therapeutic strategies, there is a growing need for well-established biomarkers and improved diagnostic and therapeutic approaches, which remain limited to date.

Platelets are the smallest blood cells and are major regulators of hemostasis and thrombosis, but they are also involved in acute and chronic inflammatory processes (6). Platelet dysfunction has been associated with several neurodegenerative diseases, including Alzheimer's and Parkinson's disease (7, 8). Platelets are a source of amyloid precursor protein (APP) and Aβ peptides in the circulation and release various Aß species upon activation (7, 9–12). Moreover, platelet activation profiles and adhesive properties are altered in AD (10, 13–15).

In aged transgenic mouse models of AD (APP23) which develop parenchymal plaques and CAA, circulating platelets are pre-activated and adhere to vascular Aβ deposits, leading to cerebral vessel occlusion (10, 13). Platelet dysfunction has also been linked to other pathological hallmarks of AD, including mitochondrial impairment (16), abnormal tau metabolism (17) and altered neurotransmitter levels (18), all of which may contribute to disease progression.

In previous studies using the APP23 mouse model, we demonstrated that platelets convert soluble Aβ40 peptides into fibrillar Aβ aggregates *in vitro* (10, 19–22). We further identified two direct platelet surface receptors for Aβ40: the fibrinogen receptor integrin αIIbβ3 and the major collagen receptor glycoprotein VI (GPVI) (19, 22). Blocking these receptors or the ADP receptor P2Y_12_ reduced Aβ aggregation *in vitro* and decreased Aβ accumulation in cerebral vessels *in vivo* (19, 22).

Several clinical studies have reported alterations in platelet parameters in AD patients. Sevush et al. (14) suggested that platelet activation contributes to AD pathogenesis reporting increased platelet aggregation, elevated P-selectin exposure and enhanced leukocyte-platelet aggregates. Stellos et al. (15) identified elevated P-selectin and integrin αIIbβ3 expression at the platelet surface and proposed platelet activation as a prognostic biomarker for cognitive decline in AD. Ramos-Cejudo et al. (23) further associated platelet phenotypes with dementia progression in AD. Other studies have identified altered platelet protein expression profiles in AD patients (24, 25). Changes in platelet APP ratios, secretase activity, and fibrinogen deposition in CAA positive vessels have also been proposed as potential biomarkers for AD (26–30).

Histological markers of thrombosis, particularly fibrinogen accumulation, have been identified in AD brains, with increased deposition observed in the cortex and hippocampus and associated with vessel enlargement and occlusion in AD patients (31, 32).

Despite, the role of platelets in AD pathology and their potential use as early diagnostic biomarker remain unclear. Robust clinical data, particularly in early AD stages, are limited. Therefore, in the present study, we examined platelet morphology and activation in patients with early-stage AD to identify disease-associated platelet phenotypes that may be useful for diagnosis and future therapeutic strategies.

## 2 Materials and methods

### 2.1 Subjects

A total of 46 patients with AD and 17 healthy elderly controls were included in the study. The diagnosis of Alzheimer's disease (AD) was based on the criteria of the National Institute of Neurological Disorders and Stroke–Alzheimer Disease and Related Disorders (NINCDS–ADRDA). The clinical severity of cognitive impairment was assessed by the CERAD plus neuropsychological battery. Patients were recruited during clinical routine diagnostic procedures of cognitive impairment. All subjects were able to give informed consent according to clinical assessment and suffered from mild AD (MMSE scores 20–26).

Age-matched controls were recruited from the local blood bank. Exclusion criteria included any form of dementia, known coagulation disorders, inherited platelet dysfunction, or any anti-platelet medication.

Fresh citrate-anticoagulated blood (105 mM Na_3_-citrate, BD-Vacutainer^®^ Becton, Dickinson and company) was obtained from all participants (>70 years of age) and processed immediately by room temperature.

Experiments with human blood were reviewed and approved by the Ethics Committee of the Heinrich-Heine-University, who approved the collection and analysis of the tissue. Subjects provided informed consent prior to their participation in the study (patients' consent: permitted ethical votes; study number 4845R, ID 2014-102828; ID 2018-140KFogU). The study was conducted in accordance with Declaration of Helsinki principles and the International Council for Harmonization Guidelines on Good Clinical Practice.

### 2.2 Flow cytometry

Flow cytometry was performed to assess platelet surface glycoproteins and activation markers, including P-selectin exposure and activated integrin α_IIb_β_3_, as described previously (33). Briefly, citrated whole blood was diluted 1:10 in human Tyrode's buffer (137 mM NaCl, 2.8 mM KCl, 12 mM NaHCO_3_, 0.4 mM NaH_2_PO_4_ and 5.5 mM glucose, pH 6.5). Blood samples were mixed with antibodies (all diluted 1:10 in a total reaction volume of 30 μl), incubated for 15 min at RT in the dark, and stimulated with indicated agonists. The addition of Dulbecco's phosphate buffered saline (DPBS) was used to stop the reaction and the samples were analyzed on a FACSCalibur flow cytometer (BD Biosciences). P-selectin exposure and active integrin α_IIb_β_3_ at the platelet surface were determined using a CD62-PE and the PAC-1-FITC antibody (PAC-1-FITC, #340507; CD62P-PE, #348107, BD Biosciences). For the analysis of surface expression of different glycoproteins as well as activation-dependent upregulation of integrin β_3_ expression, blood samples were mixed with specific antibodies [Integrin α_5_/CD49e, Tap.A12-FITC; GPIb-FITC, #348083; CD61-PE, #555754; BD Biosciences and GPVI-PE, #565241; BD Pharmingen (Heidelberg, Germany)] and incubated for 15 min at RT in the dark. For determination of APP/Aβ immunoreactivity of platelets, we used a FITC-labeled Aß antibody that detects APP and Aß at the surface of platelets.

For the flow cytometric analysis of basal platelet function in AD patients, instrument settings, including PMT voltages, gain, and thresholds, were kept constant for all measurements for both AD patients and the control cohort. A doublet discrimination for flow cytometric was not performed and represents a limitation of the flow cytometric analysis within this manuscript. Platelets were gated using their specific forward scatter and side scatter profile. Fluorescence minus one controls were used to define positive gates for all used markers.

### 2.3 Isolation of human platelets

Platelet count and mean platelet volume (MPV) in blood from AD patients and healthy controls were measured using a hematology analyzer (Sysmex KX-21N, Norderstedt, Germany). For platelet preparation, blood was centrifuged at 231 *g* for 10 min. Thereafter, the upper phase, i.e., the platelet-rich plasma (PRP), was carefully transferred into Dulbecco's phosphate buffered saline (DPBS, pH 6.5) containing apyrase (2.5 U/ml; Sigma-Aldrich, #A7646) and Prostaglandin E1 (PGE_1_, 1 μM; Sigma-Aldrich, #P5515). The DPBS PRP mixture was centrifuged at 1,000 *g* for 6 min without brakes to induce the formation of a platelet pellet. The pellet was resuspended in Tyrode's buffer (137 mM NaCl, 2.8 mM KCl, 12 mM NaHCO_3_, 0.4 mM NaH_2_PO_4_ and 5.5 mM glucose, pH 6.5). The cell count was determined using a hematology analyzer (Sysmex KX-21N, Norderstedt) and adjusted for the following experiment.

### 2.4 Transmission electron microscopy (TEM)

Non-stimulated platelets were isolated and fixed in Karnovsky's solution for 1 h at room temperature and stored at 4 °C as described previously. Briefly, for electron microscopic studies, cell pellets were embedded in agarose at 37 °C, coagulated, cut in small blocks, fixed in Karnovsky's solutions, postfixed in osmium tetroxide, and embedded in glycid ether and cut using an ultramicrotome (Ultracut Reichert, Vienna, Austria). Ultrathin sections (30 nm) were mounted on copper grids and analyzed using a Zeiss LIBRA 120 transmission electron microscope (Carl Zeiss, Oberkochen, Germany) (10).

The OCS surface area was quantified as a percentage of total platelet area using >14 images per subject. Images were randomly selected and include a min. of three platelets/image.

### 2.5 Statistical analysis

Statistical analyses were performed with GraphPad Prism (Prism 9; Graph Pad Software, Inc.). Unpaired *t*-test or two-way ANOVA were applied as appropriate. For patient data, check for normality was performed using the Shapiro–Wilk test. Statistical differences between two groups were determined using an unpaired multiple *t*-test or a two-tailed unpaired *t*-test for non-parametic data. For the analysis between more than two groups, an ordinary two-way ANOVA with a Sidak's multiple comparison *post-hoc* test was used. The indicated sample size for each experiment reflects the number of independent biological replicates. Data are presented as mean ± SEM, and *p* values < 0.05 were considered statistically significant (^*^p < 0.05, ^**^p < 0.01).

## 3 Results

### 3.1 Normal platelet count and size and unaltered glycoprotein expression but increased APP/Aß binding to platelets from AD patients

**Figure 1:**
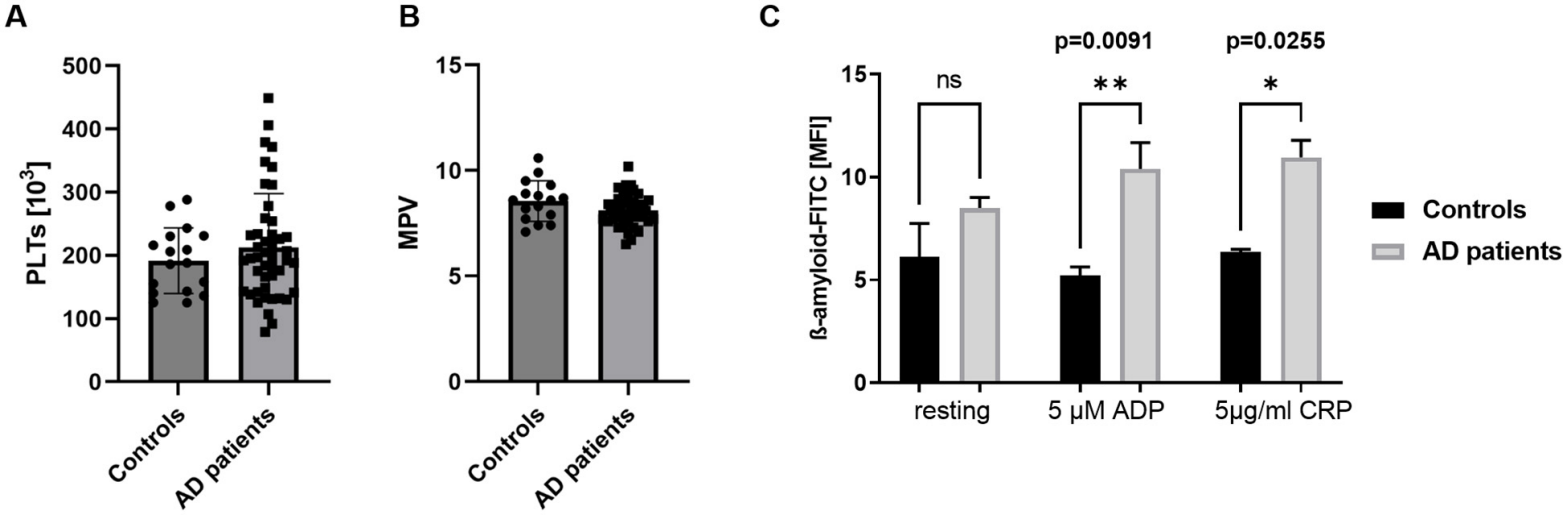
Normal platelet count and size but increased Aß binding to platelets from AD patients. **(A)** Platelet counts and **(B)** MPV (platelet size) in AD patients and age-matched controls (*n* = controls 17, patients 46). **(C)** Binding of Aß to platelets under resting (non-stimulating) and activated conditions was determined by flow cytometry using a FITC-labeled antibody against Aß and APP. Different agonists were used to induce the activation of different signaling pathways in platelets. Results are represented as MFI. Bar graphs indicate mean values ± SEM. Statistical analyses were performed using a multiple unpaired *t*-test **(A, B)** and a two-way ANOVA with Sidak's multiple comparison test **(C)**. *p < 0.05; **p < 0.01. ADP, adenosinediphosphate; CRP, collagen-related peptide; MPV, mean platelet volume; MFI, mean fluorescence intensity.

In this study, platelets from male and female patients with AD were analyzed and compared with age-matched healthy controls. As shown in Supplementary Figure S1A, all subjects included in this study were older than 70 years (Supplementary Figure S1A). Platelet count and size, expressed as mean platelet volume (MPV), were measured in blood samples from AD patients and healthy controls using a hematology analyzer (Sysmex KX-21N, Norderstedt). As shown in Figures 1A, B, no differences in platelet count or MPV were observed between AD patients and age-matched controls (Figures 1A, B). Sex-specific analyses likewise revealed no differences in platelet account or MPV (Supplementary Figures S1B, C) indicating that platelet number and size were comparable between AD patients and healthy controls as well as between female and male AD patients. Clinical parameters of the here analyzed AD patient cohort is summarized in Supplementary Table S1.

Next, we analyzed APP/Aβ immunoreactivity at the platelet surface by assessing the binding of Aß to platelets under resting (non-stimulating) and activated conditions. Platelets were activated using ADP, which stimulates the ADP receptors P2Y1 and P2Y12 and CRP, which activates the major collagen receptor on the platelet surface, GPVI. As shown in Figure 1C, no differences in APP/Aß binding were observed under resting conditions. However, upon platelet activation, significantly increased binding of the anti-APP/Aß antibody was detected on platelets from AD patients compared with those from healthy controls.

**Figure 2:**
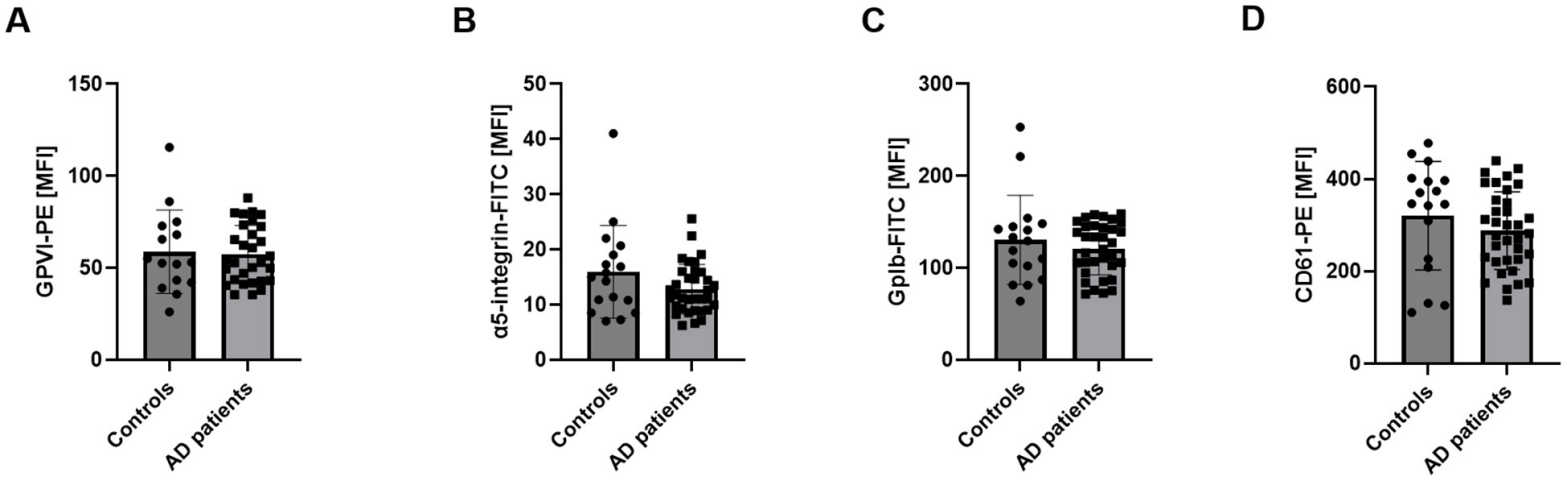
Unaltered glycoprotein expression at the surface of platelets from AD patients. Glycoprotein exposure at the platelet surface was determined by flow cytometry using different antibodies against **(A)** GPVI, **(B)** α5-integrin, **(C)** GPIb and **(D)** CD61 (subunit of integrin αIIbβ3). Results are represented as MFI. Bar graphs indicate mean values ± SEM. Statistical analyses were performed using a two-tailed unpaired *t*-test. *N* = 17 (controls), 35 (patients). MFI, mean fluorescence intensity.

To determine whether increased glycoprotein expression accounts for enhanced APP/Aß binding observed in AD platelets, we analyzed platelet glycoprotein expression by flow cytometry. No differences were detected. Surface exposure of GPVI, α5-integrin, GPIb and CD61 on resting platelets was comparable between AD patients and age-matched healthy controls (Figures 2A–D). Sex-specific analyses also revealed no differences in glycoprotein expression between male and female AD platelets (Supplementary Figures S2A–D).

### 3.2 Transmission electron microscopy revealed differences in the number of granules in platelets from AD patients

**Figure 3:**
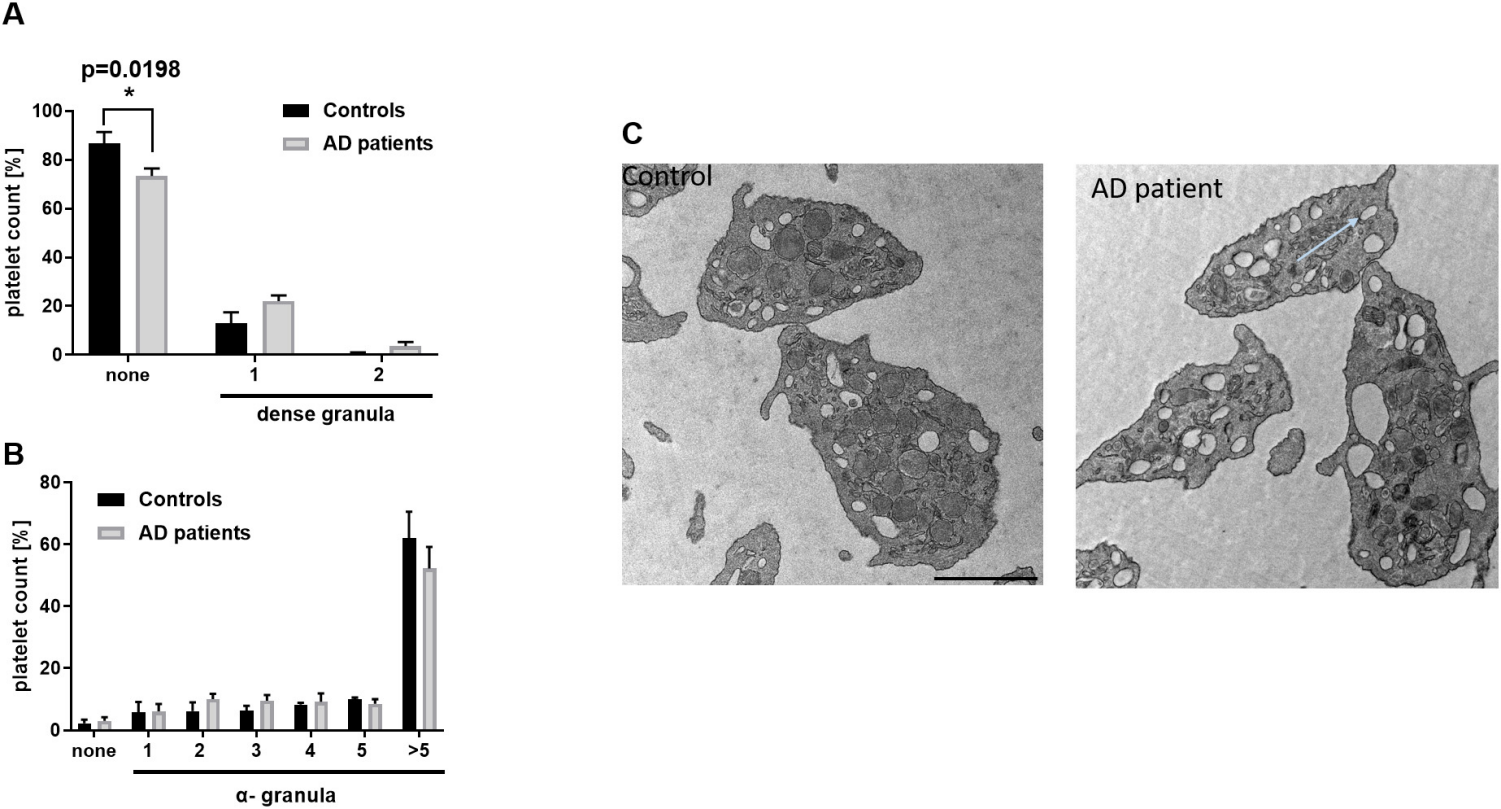
Different number of granules in platelets from AD patients. Analysis of the number of **(A)** dense and **(B)** alpha granules in platelets from AD patients and healthy subjects by transmission electron microscopy. **(C)** Representative images are shown. Scale bar 1 μm, Statistical analyses were performed using a two-way ANOVA with Sidak's multiple comparison test. *p < 0.05. *N* = 4–5.

To analyze platelet morphology in more detail, transmission electron microscopy (TEM) was performed. First, the number of dense and alpha granules in platelets from AD patients was quantified and compared with healthy controls. As shown in Figure 3, significantly fewer platelets from AD patients displayed no dense granules whereas no differences were detected in platelets containing one or two dense granules per section (Figures 3A, C). These findings indicate that platelets from AD patients contain more dense granules than those from healthy individuals.

In addition, the number of alpha granules was analyzed. No differences were observed between platelets from AD patients and healthy controls. The majority of platelets from both groups contained more than 5 alpha granules per platelet, as assessed by TEM (Figures 3B, C).

### 3.3 Significantly increased open canalicular system in platelets from AD patients

**Figure 4:**
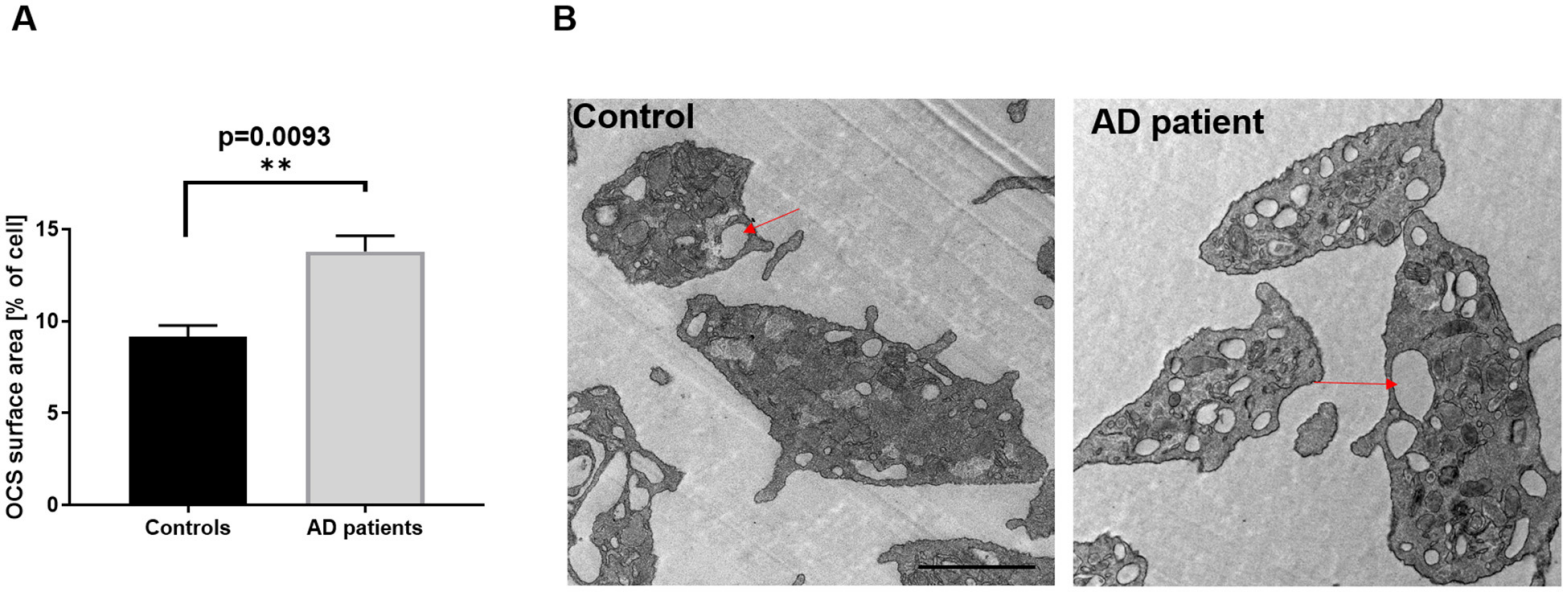
Significantly increased open canalicular system in platelets from AD patients. The area of the open canalicular system of platelets has been analyzed by transmission electron microscopy. Quantification of OCS surface area as percentage of the total cell area is shown. **(A)** Bar graph depicts mean values ± SEM. **(B)** Representative images from transmission electron microcopy. Scale bar 1 μm. Statistical analyses were performed using a two-tailed unpaired *t*-test. **p < 0.01. *N* = 3–5.

In a second approach, the open canalicular system (OCS) of platelets was investigated. Ultrastructural analysis by TEM revealed a significantly increased OCS area in platelets from AD patients compared with those from healthy controls (Figures 4A, B). TEM images demonstrated an elevated OCS surface area, expressed as percentage of total platelet area, in AD platelets (Figure 4B). These results suggest that detailed morphological analyses, including granule number and OCS size, may be promising for the identification of prognostic biomarkers for AD.

### 3.4 No major differences in the activation profile of platelets from AD patients

**Figure 5:**
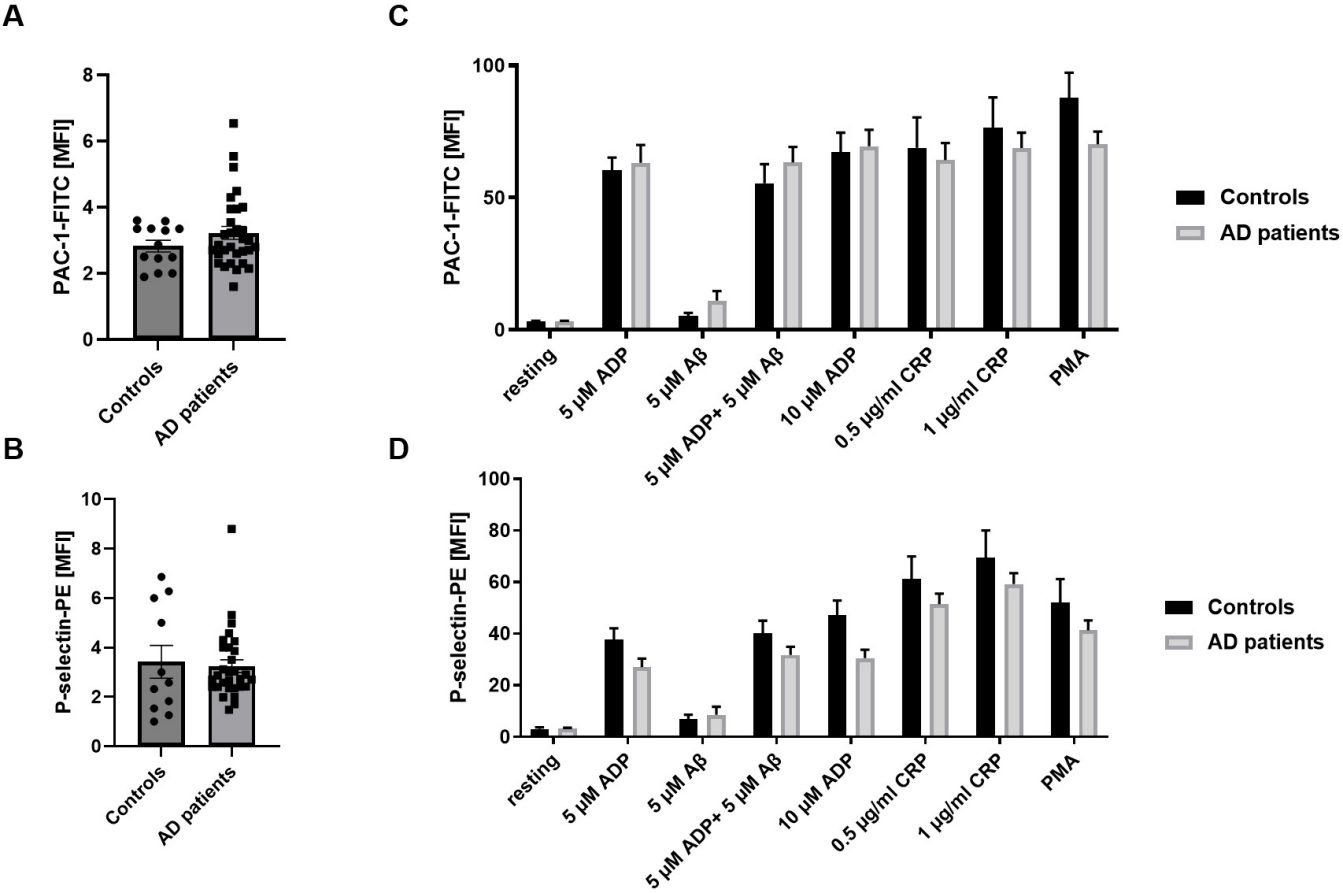
Platelets from AD patients show no major differences in platelet integrin activation and degranulation. **(A)** Active integrin (PAC-1 binding to integrin αIIbβ3) and **(B)** platelet degranulation (P-selectin-PE) under resting conditions were determined by flow cytometry using whole blood from AD patients and age-matched controls (*n* = 11) (controls) and 30 (patients). **(C, D)** Active integrin and P-selectin exposure under activated conditions using different agonists to stimulate different signaling pathways in platelets. Data are represented as MFI. Bar graphs indicate mean values ± SEM. Statistical analyses were performed using a two-tailed unpaired *t*-test **(A, B)** and a two-way ANOVA with Sidak's multiple comparison test **(C, D)**. *p < 0.05. ADP, adenosinediphosphate; CRP, collagen-related peptide; Aß, amyloid-beta; PMA, Phorbol-myristate-acetate; MFI, mean fluorescence intensity. *N* = 11 (controls), 30 (patients).

To compare platelet activation profiles between AD patients and age-matched controls, we determined surface expression of active integrin αIIbβ3 and P-selectin. P-selectin exposure serves as marker of alpha-granule degranulation, whereas active integrin αIIbβ3 mediates fibrinogen binding and is critical for platelet aggregation and thrombus formation. Under non-stimulating (resting) conditions, no differences in integrin activation or P-selectin exposure were observed between the two groups (Figures 5A, B).

Platelet activation was then analyzed following stimulation with different agonists that activate distinct signaling pathways in platelets. Low dose ADP, Aß, CRP, and PMA induced integrin activation (Figure 5C) and P-selectin exposure (Figure 5D); however, no differences between AD patients and controls were detected. A trend to reduced P-selectin exposure was observed after stimulation with 5 and 10 μM ADP in platelets from AD patients (Figure 5D). No differences were detected in the up-regulation of integrin αIIbβ3 at the platelet surface between AD patients and healthy controls (Supplementary Figures S3A). Overall, no major differences in platelet activation were observed between AD patients and healthy controls (Figures 5A–D).

### 3.5 Sex-specific alterations provide evidence for reduced integrin activation of female platelets isolated from AD patients

**Figure 6:**
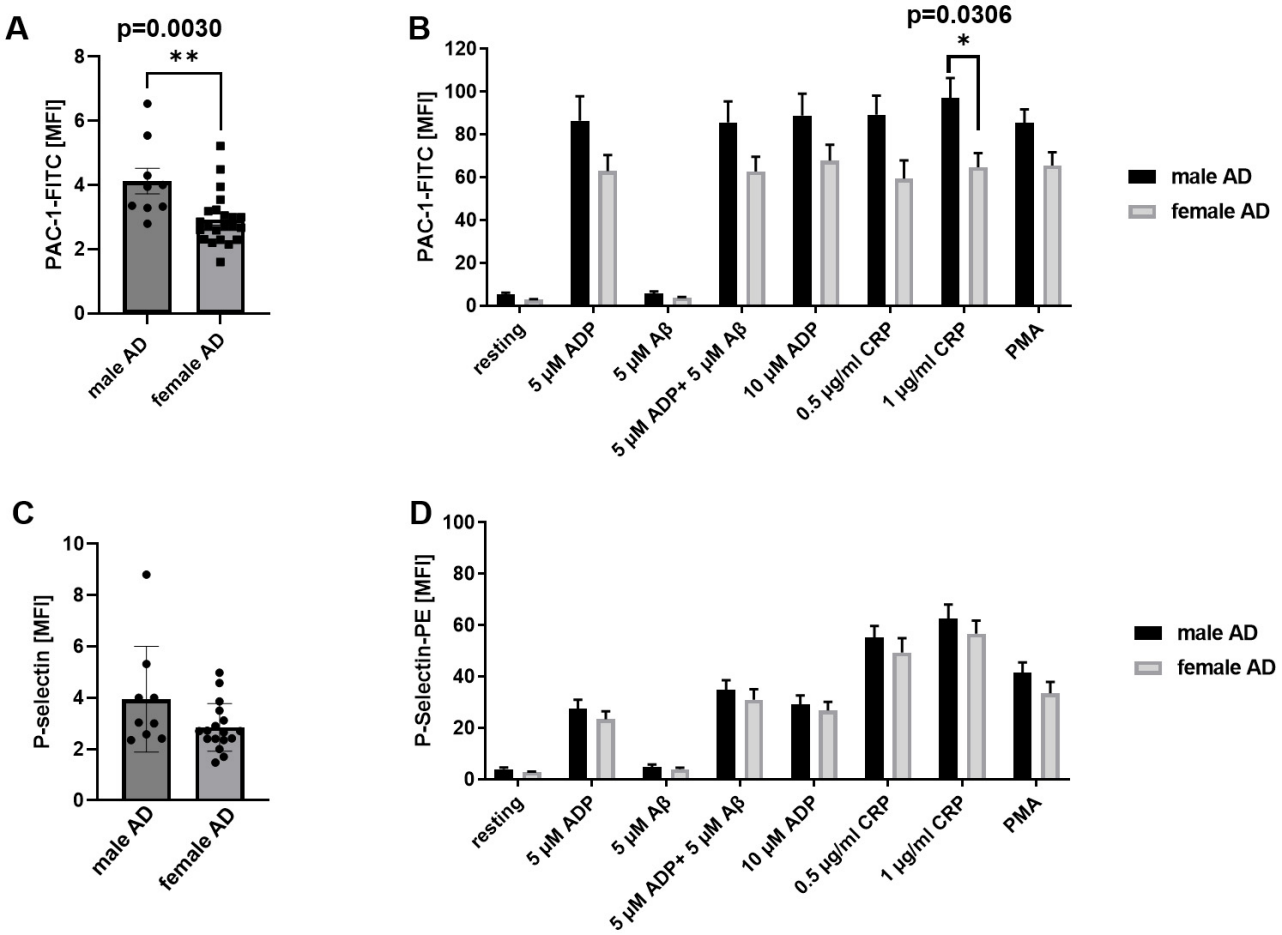
Sex-specific analysis of platelet activation revealed a reduced activation profile in female patients. **(A, B)** Active integrin αIIbβ3 (PAC-1 binding) and **(C, D)** P-selectin exposure as marker for degranulation were determined by flow cytometry. Data are represented as MFI. Bar graphs indicate mean values ± SEM. Statistical analyses were performed using a multiple unpaired *t*-test **(A, C)** and a two-way ANOVA with Sidak's multiple comparison test **(B, D)**. *p < 0.05. ADP, adenosinediphosphate; CRP, collagen-related peptide; Aß, amyloid-beta; PMA, Phorbol-myristate-acetate; MFI, mean fluorescence intensity. *p < 0.05, **p < 0.01. *N* = 9 (males), 21 (females).

Finally, we investigated potential sex-specific differences in platelet function in AD patients. As shown in Figures 6A, B, significantly reduced integrin activation was detected in platelets from female AD patients compared with male AD patients following stimulation of the collagen receptor GPVI. Notably, significant differences were also detected under resting conditions with platelets from female AD patients exhibiting lower basal integrin activation (Figure 6A). In contrast, no sex-specific differences were observed in P-selectin exposure (Figures 6C, D). Glycoprotein expression (Supplementary Figure 2) and agonist-induced up-regulation of integrin αIIbβ3 at the platelet surface were also comparable between male and female AD platelets (Supplementary Figure S3B). These findings suggest that sex-specific analyses of platelet activation profiles may be important for identifying platelet alterations and developing prognostic markers for AD.

## 4 Discussion

AD is a multifactorial disorder in which multiple pathophysiological events contribute to disease initiation and progression (3, 34). A key role for platelets in the progression of AD including CAA has been suggested by several groups (13, 15, 22, 35). Platelets store Aβ peptides and are able to release them upon platelet activation (10–12). Platelet dysfunction has been associated with AD and may contribute disease pathology during both initiation and progression (14, 15, 29, 35).

With the recent development of amyloid-antibody-based therapies for AD, a comprehensive analysis of platelet activation and its contribution to disease pathology has become increasingly important, particularly for the development of effective blood-based screening tools. Therefore, platelets may represent valuable biomarkers for the diagnosis and prognosis of AD.

Consistent with the finding of the present study, several previous studies reported unaltered platelet counts and size in AD patients, suggesting unchanged platelet turnover (14, 36). In contrast, Inestrosa et al. reported increased platelet counts in AD patients (37).

Regarding platelet activation, APP processing and protein expression in AD patients, clinical studies have yielded controversial results. Sevush et al. (14) reported elevated CD62P (P-selectin) surface expression, increased platelet aggregation and enhanced platelet-leukocyte formation. Järemo et al. (38) demonstrated increased levels of soluble P-selectin in AD patients, although no changes were detected at the platelet surface. Notably, this study included 23 patients with moderate AD. In contrast, Stellos et al. (15) observed significantly higher baseline expression of activated integrin αIIbβ3 and P-selectin in AD patients with rapid cognitive decline compared with those exhibiting slow cognitive decline.

In the present study, platelet activation under resting and stimulated conditions was largely unaltered in patients at early stages of AD. However, when samples were stratified by sex significant differences emerged between male and female AD patients. Specifically, integrin αIIbβ3 activation, as assessed by PAC-1 binding was significantly higher in platelets from male AD patients compared with female AD patients, both under resting conditions and upon CRP stimulation. These findings suggest that platelets from male AD patients are more susceptible to activating stimuli. Importantly, no such differences were detected when male and female were analyzed together, highlighting the importance of sex-specific analyses. This finding may be in accordance with other studies indicating sex differences of AD biomarkers, such as tau-protein and GFAP (39). Moreover, discrepancies between studies may be explained by differences in disease stage at the time of analysis, which is often insufficiently reported. In addition, stratification based on the rate of cognitive decline, as demonstrated by Stellos et al. (15), may be crucial for understanding platelet alterations in AD.

Additional platelet parameters have been reported in AD. For example, the number of coated platelets has been shown to be increased at early stages of AD and decreased at later stages (40), further underscoring the need to consider disease stage when analyzing platelet-related parameters. Interestingly, a study by Kumar et al. reported increased accumulation of neurotransmitter in platelets from female AD patients (18), providing further support for the necessity of sex-specific analyses to obtain reliable biomarker data.

Several studies propose platelet alterations as prognostic biomarkers in AD. Stellos et al. (15) demonstrated differences in platelet activation between patients with distinct rates of cognitive decline. Ramos-Cejudo et al. (23) suggested that increased platelet aggregation may have prognostic value in AD. In addition, changes in platelet protein expression have been proposed as potential biomarker. Yu et al. (24, 25) reported increased protein expression of PHB, GPIbα, and FINC alongside reduced ADAM10 protein expression, in platelets from AD patients. Similarly, Fu et al. observed decreased ADAM10 expression and increased adenosine A2 receptor expression in AD platelets (27). Other studies have suggested altered APP protein ratios in platelets as potential biomarkers (26, 30). Mitochondrial dysfunction in platelets has also emerged as promising candidate; Fišar et al. (41) proposed mitochondrial impairment as a primary contributor to AD progression, which is consistent with our own data showing that mitochondrial dysfunction promotes platelet-mediated Aß aggregate formation *in vitro* (21).

In the present study, we demonstrate that APP/Aß detection at the platelet surface by flow cytometry may represent a promising biomarker, as we observed markedly increased binding of an anti-APP/Aß antibody to platelets from AD patients. Additionally, platelet activation parameters-particularly in platelets from female AD patients- may serve as valuable biomarker for early AD diagnosis in women. These parameters can be readily assessed by flow cytometry. A more sophisticated, albeit technically demanding, approach is the ultrastructural analysis of platelet morphology by TEM. Here, we detected a significantly increased OCS surface area, expressed as a percentage of total platelet area, in platelets from AD patients.

Beyond its potential as a biomarker, the functional relevance of an expanded OCS in AD warrants further investigation. The OCS is an internal membrane system consisting of a network of surface-connected channels, first described more than five decades ago (42). However, the regulation and precise functional role of the OCS remain incompletely understood. Structural abnormalities of the OCS have been described in certain platelet disorders; however, how these alterations contribute to changes in platelet function is still largely unclear.

Taken together, it is crucial to (i) elucidate how platelets contribute to AD pathology in patients and (ii) develop a comprehensive panel of platelet-derived parameters that may serve as accessible blood-based biomarkers for early AD prognosis. Such a panel could include platelet activation status, APP/Aβ immunoreactivity, and OCS dimensions assessed by TEM.

### 4.1 Limitations, gaps and future recommendations

The results presented here provide evidence for alterations in platelet morphology including granule numbers and OCS structure, as well as sex-specific differences in platelet activation in AD patients. However, this study has several limitations: the OCS was quantified on 2D TEM sections. Since the OCS is a 3D tortuous network we cannot exclude that there have been small errors arising from the here used method for the detection of the OCS total surface. Furthermore, this study includes a relatively small sample size and there is a lack of a longitudinal follow-up, which may limit generalizability and introduce bias. Furthermore, an unequal number of patients and healthy controls were included into the study without effect size expectations or sample size calculation. No correction for multiplicity was performed. Therefore, future studies with larger cohorts and longitudinal design are therefore required to validate these findings and to further investigate platelet activation and morphology across different stages of AD.

## Data Availability

The original contributions presented in the study are included in the article/Supplementary material, further inquiries can be directed to the corresponding author.
