# Supplementary material for: Platelets from early-stage Alzheimer patients show enhanced amyloid binding, an elevated open canalicular system and sex-specific differences in their activation profile
Source: Front Neurol. 2026 Mar 19;17:1759268. doi: 10.3389/fneur.2026.1759268 (PMC13043387; doi:10.3389/fneur.2026.1759268)
Supplement: Supplementary file 1 [file Data_Sheet_1.pdf]

Supplementary Material

1 Supplementary Figures and Tables

1.1 Supplementary Figures

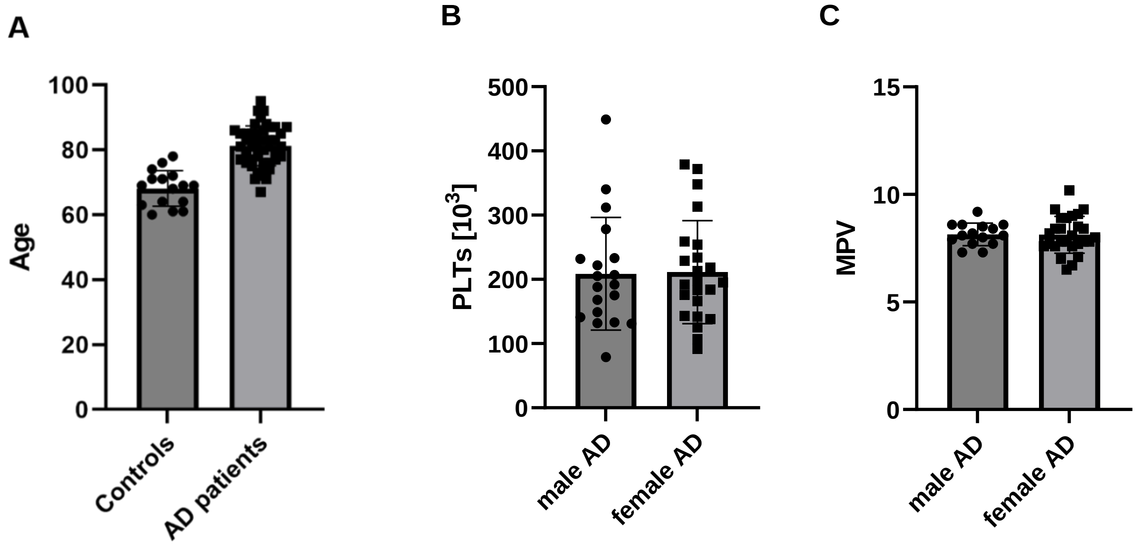

Supplementary Figure 1. Overview of age, platelet counts and size of AD patients and healthy subjects included in this study. (A) All subjects were >70 years of age. (B) Platelet counts and (C) MPV of platelets from male and female AD patients. Bar graphs indicate mean values ± SEM. Statistical analyses were performed using a two-tailed unpaired t-test. N = controls 17 and patients 46.

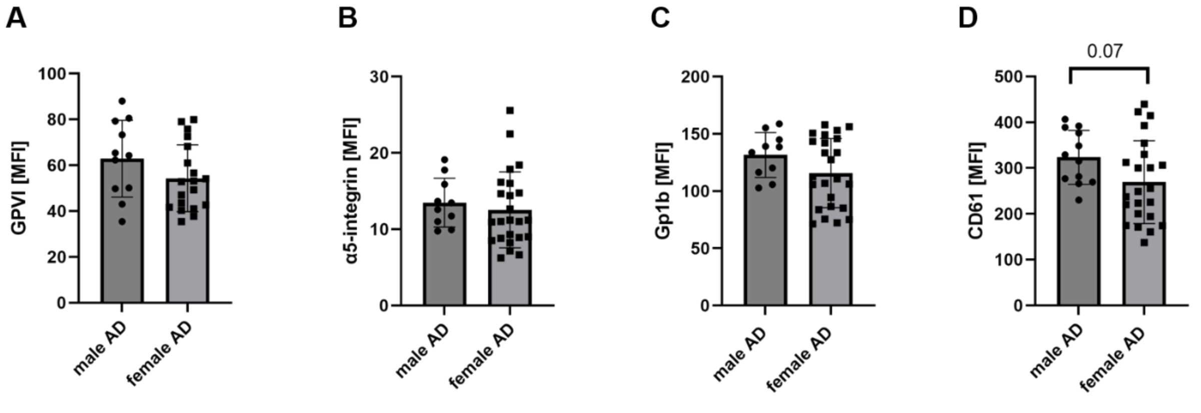

Supplemental Figure 2. No differences in glycoprotein expression at the platelet surface between female and male AD patients. Glycoprotein exposure at the platelet surface was determined by flow cytometry using different antibodies against (A) GPVI, (B) a5-integrin, (C) GPIb and (D) CD61 (subunit of integrin aIIbb3. Bar graphs indicate mean values ± SEM. Statistical analyses were performed using a two-tailed unpaired t-test. N = 9 (males), 21 (females).

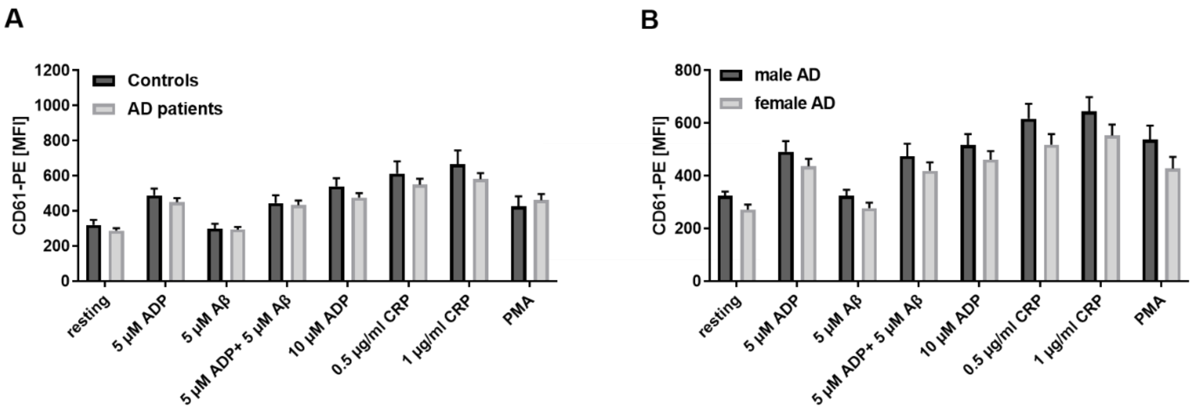

Supplemental Figure 3. No differences in the exposure of integrin αIIbβ3 at the platelet surface of AD patients. The exposure of integrin αIIbβ3 under resting and under stimulating conditions was determined by flow cytometry using a CD61 antibody. No differences were detected between AD patients and healthy controls (A) as well as between male and female platelets from AD patients (B). Data are represented as MFI. Bar graphs indicate mean values ± SEM. Statistical analyses were performed using a multiple unpaired t-test. ADP, adenosinediphosphate, CRP, collagen-related peptide, Aß, amyloid-beta, PMA, Phorbol-myristate-acetate, MFI, mean fluorescence intensity. (A): N = 17 (controls), 35 (patients). B: N = 9 (males), 21 (females).

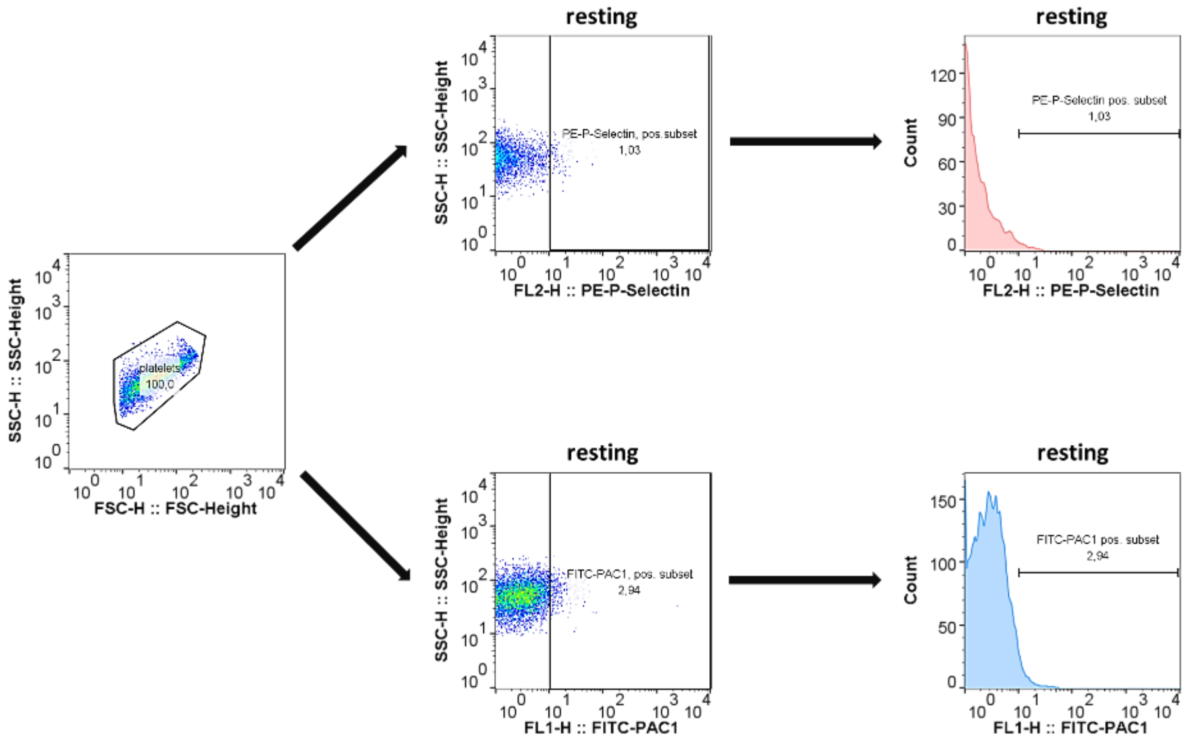

Supplemental Figure 4. Gating strategy for flow cytometric analysis of AD patients. Gating strategy for the analysis of platelet activation using whole blood from AD patients. Whole blood samples were first gated on platelets according to their specific FSC/SSC profile. Representative histograms for P-selectin and PAC1 binding to the platelet surface after gating is provided.
