# Supplementary material for: Platelets from early-stage Alzheimer patients show enhanced amyloid binding, an elevated open canalicular system and sex-specific differences in their activation profile
Source: Front Neurol. 2026 Mar 19;17:1759268. doi: 10.3389/fneur.2026.1759268 (PMC13043387; doi:10.3389/fneur.2026.1759268)
Supplement: Supplementary file 2 [file Table_1.docx]

**Table S1. Clinical parameter of Alzheimer patients**. Continuous data are represented as mean ± SEM. Dichotomous data are represented as absolute frequency with percentage (%).

|  | Age | Female  sex | Male sex | CHD | T2D | SAPT | Memantin | aHT | Cancer | Hypothyroidism |  |
| --- | --- | --- | --- | --- | --- | --- | --- | --- | --- | --- | --- |
| AD  (n=46) | $81$  ±0.91 | $33$ | 13 | $9$ | $8$ | 17 | 4 | 23 | 3 | 8 |  |
|  |  |  |  |  |  |  |  |  |  |  |  |

AAA = abdominal aortic aneurysm; aHT = arterial hypertension; CHD = coronary heart disease; AT = Arterial thrombi (thrombectomy patients); SAPT = single anti-platelet therapy; T2D: type 2 diabetes.

Control whole blood samples were collected from anonymized healthy donors (n=17; average age: 68.1 ± 1.34 years) from the blood bank of the University Hospital Duesseldorf. Exclusion criteria for control subjects were as follows: Any form of dementia, known coagulation disorders, inherited platelet dysfunction, or any anti-platelet medication.
